# Hesperidin Supplementation Enhances Neuroprotection by Modulating the ERK/MAPK Pathway and Tau Immunoreactivity in an AlCl3‐Induced Cognitive Impairment Model Using Wistar Mice

**DOI:** 10.1002/alz70855_096954

**Published:** 2025-12-23

**Authors:** Rademene Oria, Runyi Bassey Ben, Cynthia N. Tangban

**Affiliations:** ^1^ University of Cross River State, Cross River, Cross River, Nigeria

## Abstract

**Background:**

Alzheimer's disease (AD) is a rampant neurodegenerative disorder, characterized by progressive cognitive impairment and considerable neuropathological changes. Hesperidin, a flavonoid glycoside predominantly found in citrus fruits is known for its neuroprotective properties. There is an information gap on the effect of hesperidin on AlCl3 induced model of cognitive impairment similar to AD.

**Methods:**

Twenty‐eight male albino Wistar mice were divided into four groups, each with seven randomly selected and administered with AlCl3(100mg/kg po) or co‐administered with zingerone (100mg/kg po) for 60days. After administration, morris water maze and Y‐maze tests were carried out to evaluate cognition. Biochemical investigation to access neuroinflammation was performed via measures of TNF‐α and IL‐1β. Furthermore, immunohistochemical assessment of the expression of ERK, IBA1 and Tau in the hippocampus and prefrontal cortex was carried out.

**Result:**

Results indicated that AlCl3 only induced cognitive impairment, increased tissue levels of TNF‐α and IL‐1β along with upregulation of ERK, Tau and increase in IBA1‐positive microglia. However, treatment with hesperidin restored these parameters to levels comparable to the control group

**Conclusion:**

Hence, Hespiridin is a promising candidate to be included into the search for safe and effective anti‐AD molecules.

